# Diagnostic Ion-Guided Isolation and Characterization of Trace Periplocin-Derived Cardenolide Metabolites from Rat Urine

**DOI:** 10.3390/molecules31142436

**Published:** 2026-07-11

**Authors:** Peng Zhao, Fanjiao Zuo, Caixia Li, Haoran Wu, Yingjing Zhao, Yanjin Li, Yameng Zhu, Ye Shang, Liqin Ding, Jun He

**Affiliations:** State Key Laboratory of Chinese Medicine Modernization, Tianjin University of Traditional Chinese Medicine, Tianjin 301617, China

**Keywords:** periplocin, *Periplocae Cortex*, cardenolide metabolites, diagnostic ion, UPLC-Q-TOF-MS/MS, targeted isolation, gomphogenin, 17*α*-asclepioside

## Abstract

Cardiac glycosides from *Periplocae Cortex* are bioactive constituents, but isolating and structurally confirming their trace in vivo metabolites remains challenging because authentic standards are often unavailable. We developed a steroid-core fragment-guided strategy based on the diagnostic ion at *m*/*z* 355.2270 that integrates metabolite screening, targeted purification, and structural characterization. Periplocin-related analytes were detected in rat urine and plasma after oral periplocin administration using HPLC-QQQ-MS/MS and UPLC-Q-TOF-MS/MS. Guided by the diagnostic ion, 13 L of rat urine was fractionated using macroporous resin, Sephadex LH-20, ODS chromatography, and preparative HPLC. Two trace metabolites were isolated and identified as gomphogenin (M1, 4 mg) and 17*α*-asclepioside (M2, 8 mg) by HR-MS and NMR spectroscopy. Periplocin, periplogenin, and periplocymarin were confirmed using reference standards. Structural comparison supported a putative transformation pathway involving deglycosylation and steroid-core modification. In a hypoxia/reoxygenation-injured H9c2 cell model, M1 and M2 increased cell viability at 50 μM, supporting further evaluation using complementary cellular endpoints. This diagnostic-ion-guided workflow enables the targeted isolation of trace cardenolide metabolites from biological matrices and provides reference compounds for future pharmacokinetic and pharmacological studies.

## 1. Introduction

Cardiac glycosides have long been used in the treatment of heart disease [1]. *Cortex Periplocae*, the dried root bark of *Periploca sepium* Bge., is a representative botanical source of these compounds and has been used in Traditional Chinese Medicine for chronic heart failure and rheumatoid arthritis. Periplocin (C_36_H_56_O_13_), a typical cardiotonic steroid, is considered a major bioactive cardiac glycoside and a primary quality marker of *Cortex Periplocae* [2,3]. Previous studies have reported multiple biological activities of periplocin, including cardiotonic [4,5], antitumor [6,7,8], and anti-rheumatic effects [9]. Among these activities, its cardiovascular effects are particularly relevant to the traditional use of *Cortex Periplocae* [10]. Periplocin has been reported to enhance myocardial contractility, slow heart rate, improve left ventricular structure and function, and increase SERCA mRNA expression in rats with chronic heart failure [11,12,13,14].

After administration, cardiac glycosides may undergo extensive in vivo biotransformation, and their pharmacological effects cannot always be attributed solely to the parent compound. This issue is especially important for periplocin because its therapeutic effects, systemic exposure, and safety profile may be influenced by structurally related metabolites formed after oral administration. Periplocymarin and periplogenin have been identified as metabolites of periplocin in rat plasma [15]. Periplocymarin exhibits marked cardiotonic activity [16,17,18] and shows higher in vivo exposure than periplocin [19], while periplogenin has been associated with protective effects in cardiovascular-related conditions [20] and anti-inflammatory activity [21,22]. These findings indicate that periplocin metabolites may contribute to the pharmacological material basis of *Cortex Periplocae*. However, many in vivo metabolites occur at trace levels and are difficult to isolate and structurally confirm because of their low abundance, matrix interference, and the limited availability of authentic reference standards. Therefore, improved analytical and preparative strategies are needed for the targeted detection, purification, and structural confirmation of periplocin-related metabolites from biological matrices.

Mass spectral fragmentation provides important structural information for natural product analysis [23]. Fragment ion-based strategies have been widely used to assign diagnostic fragments, infer fragmentation pathways, and recognize structurally related natural products in complex samples [24,25,26,27]. Liquid chromatography coupled with high-resolution mass spectrometry (LC-HRMS) is particularly useful for metabolite profiling in biological matrices because it provides accurate mass measurements and product-ion information. For cardiac glycosides, the conserved steroid nucleus offers a common structural feature for targeted screening. Under electrospray ionization and collision-induced dissociation, compounds sharing this cardenolide core may generate characteristic fragment ions derived from the steroid nucleus. Diagnostic ion filtering can therefore reduce endogenous interference and facilitate the detection of trace metabolites with related steroid-core structures in complex biological samples [28].

Accordingly, selective analytical strategies capable of detecting, isolating, and structurally confirming trace periplocin-related metabolites are needed to clarify their potential contribution to the pharmacological material basis of *Cortex Periplocae*. In this study, we established a steroid core-guided targeted strategy using UPLC-Q-TOF-MS/MS. The diagnostic ion at *m*/*z* 355.2270 was used to screen periplocin-related metabolites in rat urine and to guide their enrichment and purification from large-volume urine samples. Unlike previous metabolite profiling studies that relied mainly on LC-MS identification, this work integrates diagnostic ion-guided screening, targeted purification, and structural confirmation, enabling the isolation of trace metabolites directly from biological matrices. Using this workflow, two trace metabolites, gomphogenin (M1) and 17*α*-asclepioside (M2), were isolated and structurally confirmed. Their effects on cell viability were further evaluated in a hypoxia/reoxygenation-induced H9c2 cell injury model as a preliminary biological assessment.

## 2. Results and Discussion

### 2.1. Steroid Core-Guided Screening of Periplocin Metabolites in Biological Samples

**Figure 1:**
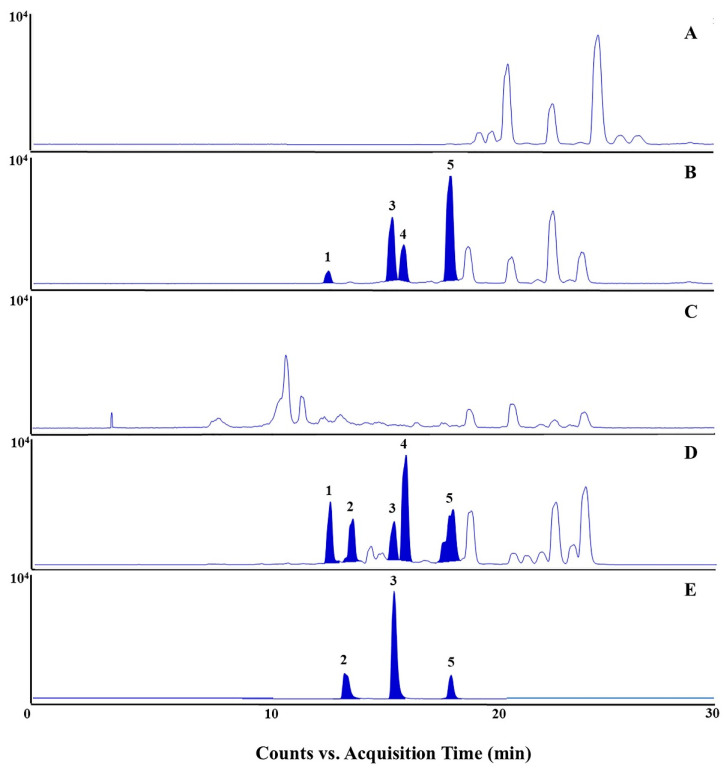
The MRM chromatograms of five analytes: (**A**) blank plasma sample, (**B**) plasma sample after oral administration of periplocin, (**C**) blank urine sample, (**D**) urine sample after oral administration of periplocin, and (**E**) mixed standard sample. 1. gomphogenin (M1), 2. periplocin, 3. periplogenin, 4. 17*α*-asclepioside (M2), 5. periplocymarin.

To establish a targeted strategy for discovering periplocin-related metabolites, blank biological samples were compared with urine and plasma samples collected after oral administration of periplocin. In the HPLC-QQQ-MS/MS analysis, a steroid-core-related transition from *m*/*z* 391.3 to *m*/*z* 355.2 was used to monitor analytes capable of generating the conserved cardenolide fragment after glycosidic cleavage and dehydration. Five analyte peaks were detected in the dosed samples, whereas no corresponding signals were observed in the blank samples (Figure 1). Peaks 2, 3, and 5 were assigned as periplocin, periplogenin, and periplocymarin, respectively, by comparison with authentic standards. Peaks 1 and 4 were reproducibly detected in both urine and plasma but could not be assigned using the available reference standards at the screening stage; therefore, they were designated as M1 and M2. The HPLC-QQQ-MS/MS method showed satisfactory analytical consistency for the three reference compounds. The intra-day and inter-day precision RSDs were 2.01–5.23% and 2.42–4.81%, respectively, while the repeatability RSDs ranged from 2.33% to 3.95% (Table S1). Because the plasma and urine samples differed in their collection periods and matrix-specific pretreatment, the chromatographic responses were interpreted qualitatively. Urine was selected as the preparative matrix because its large cumulative volume (13 L) provided sufficient material for the enrichment and isolation of trace metabolites.

**Figure 2:**
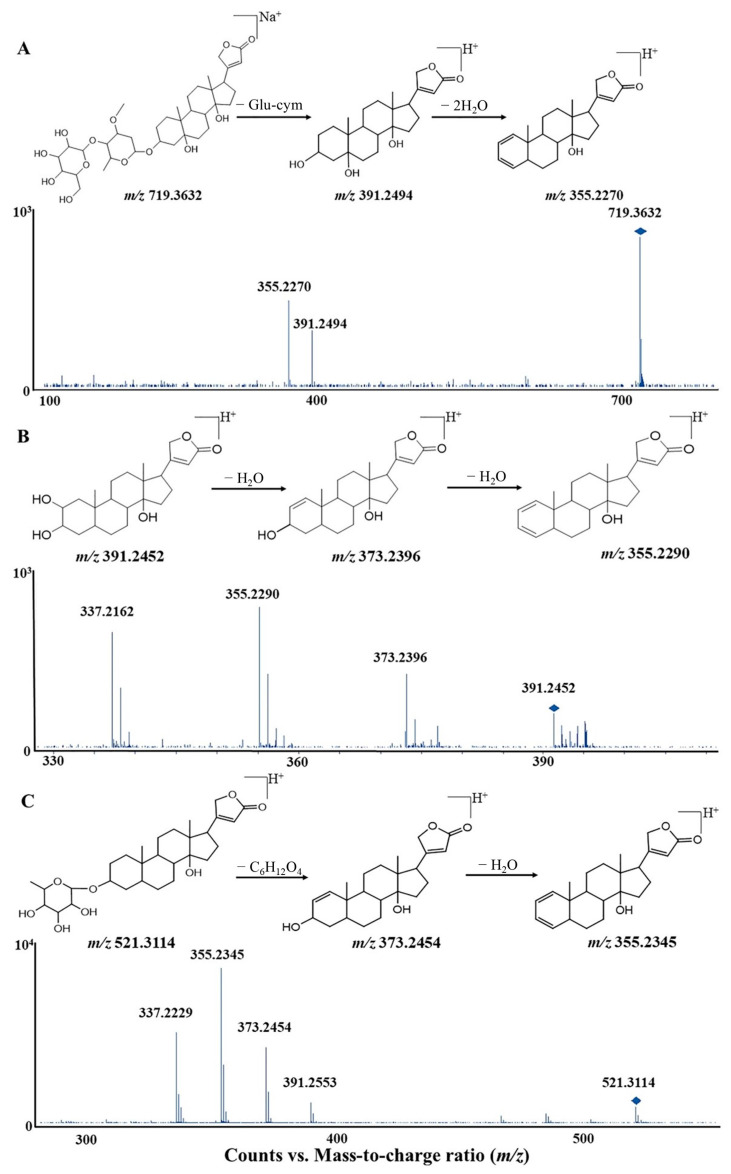
MS/MS spectra and proposed fragmentation pathways of (**A**) periplocin, (**B**) gomphogenin (M1), and (**C**) 17*α*-asclepioside (M2).

UPLC-Q-TOF-MS/MS was then used to verify the diagnostic fragmentation behavior of periplocin-related analytes. Periplocin was observed at *m*/*z* 719.3632 [M + Na]^+^, and its MS/MS spectrum showed fragment ions at *m*/*z* 391.2494 [M + Na − Glu − Cym]^+^ and *m*/*z* 355.2270 [M + Na − Glu − Cym − 2H_2_O]^+^. The abundant fragment ion at *m*/*z* 355.2270 was selected as a class-informative marker because it corresponded to the conserved dehydrated cardenolide scaffold formed through glycosidic cleavage and sequential water loss. M1 and M2 generated corresponding characteristic ions in their MS/MS spectra. M1 showed a protonated molecular ion at *m*/*z* 391.2452 [M + H]^+^, corresponding to the molecular formula C_23_H_34_O_5_ (calcd for [M + H]^+^, *m*/*z* 391.2479), and produced the diagnostic fragment ion at *m*/*z* 355.2290 [M + H − 2H_2_O]^+^. M2 showed a protonated molecular ion at *m*/*z* 521.3114 [M + H]^+^, corresponding to the molecular formula C_29_H_44_O_8_ (calcd for [M + H]^+^, *m*/*z* 521.3109), and produced the diagnostic fragment ion at *m*/*z* 355.2345 [M + H − C_6_H_12_O_4_ − H_2_O]^+^ (Figure 2). The retention times of M1 and M2 were 10.43 and 13.54 min, respectively. Because structurally related steroidal compounds may generate similar fragments, candidate selection incorporated several complementary criteria. M1 and M2 were absent from the blank plasma and urine samples but were reproducibly detected in both matrices after periplocin administration. Their accurate precursor-ion masses and characteristic neutral-loss patterns were also consistent with periplocin-related cardenolides. Neither compound was detected in the periplocin dosing material or the corresponding procedural blanks. The diagnostic ion was subsequently used to track target-containing fractions during purification, and the chemical structures of M1 and M2 were established using combined HR-MS and NMR evidence. Collectively, their post-dose occurrence and structural relationship to periplocin support their assignment as putative periplocin-related metabolites.

### 2.2. Diagnostic Ion-Guided Isolation and Structural Confirmation of M1 and M2 from Rat Urine

The diagnostic ion at *m*/*z* 355.2270 represents a dehydrated cardenolide aglycone core generated after glycosidic cleavage and the sequential neutral loss of two H_2_O molecules. In this study, this diagnostic ion was used not only as an identification marker but also as a purification guide. During macroporous resin enrichment, Sephadex LH-20 chromatography, ODS chromatography, and preparative HPLC, fractions containing both the target precursor ions and the steroid-core fragment ion were prioritized for further separation. This diagnostic ion-guided workflow reduced blind fractionation of the complex urinary matrix and enabled the enrichment and isolation of two trace metabolites, M1 and M2, from rat urine after oral administration of periplocin.

For M1, the protonated molecular ion was observed at *m*/*z* 391.2455 [M + H]^+^, supporting the molecular formula C_23_H_34_O_5_ (calcd for [M + H]^+^, *m*/*z* 391.2479). M1 showed the same nominal precursor ion as periplogenin but had a different retention time, indicating that it was a structural isomer. Its MS/MS spectrum contained the characteristic cardenolide fragment ion at *m*/*z* 355, supporting the presence of a related steroidal aglycone core. In the ^13^C-NMR spectrum, the downfield shift of C-2 and the upfield shift of C-5 compared with periplogenin indicated altered oxygenation on the steroid nucleus. The oxygenated carbon signals at C-2, C-3, and C-14, together with the butenolide-related signals at C-20-C-23, were consistent with a cardenolide aglycone. The ^1^H-NMR spectrum showed characteristic signals for H-3, H2–21, and H-22. The specific rotation of M1 was ${\left[\alpha \right]}_{D}^{25}$ 9.0 (*c* 2.0, MeOH). Based on the HR-MS, MS/MS, NMR, optical-rotation data, and comparison with published data [29], M1 was identified as gomphogenin. Gomphogenin has previously been reported as a plant-derived cardenolide, whereas its direct recovery from rat urine after periplocin administration supports its assignment here as an in vivo periplocin-related metabolite.

**Figure 3:**
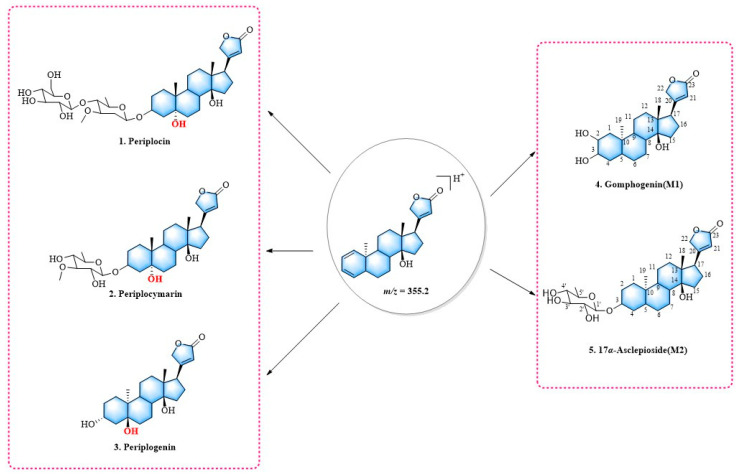
The chemical structures of five analytes.

For M2, the protonated molecular ion was observed at *m*/*z* 521.3114 [M + H]^+^, supporting the molecular formula C_29_H_44_O_8_ (calcd for [M + H]^+^, *m*/*z* 521.3109). Its MS/MS spectrum also generated the diagnostic fragment ion at *m*/*z* 355, indicating a related cardenolide skeleton. Compared with periplocymarin, the upfield shift of C-5 supported the absence of a C-5 hydroxyl group. The anomeric proton appeared at δ_H_ 5.36 (1H, d, *J* = 7.8 Hz), and the corresponding carbon signal was observed at δ_C_ 97.7. The sugar proton and carbon signals were consistent with a 6-deoxyallose moiety. Importantly, the HMBC spectrum showed a long-range correlation from H-1′ to C-3, establishing the glycosidic linkage at C-3. The HMBC correlation from H3–19 to C-5 further supported the assignment of the A/B-ring region. The specific rotation of M2 was ${\left[\alpha \right]}_{D}^{25}$ 10.0 (c 4.0, MeOH). Taking into account the HR-MS/MS data, NMR evidence, HMBC correlations, and comparison with published data [30], M2 was identified as 17*α*-asclepioside. Although 17*α*-asclepioside was originally reported from the methanolic extract of the whole plant of Asclepias fruticosa, the present study isolated and identified it from rat urine after periplocin administration. The shared cardenolide aglycone features and the diagnostic fragment ion at *m*/*z* 355 further supported the assignment of M1 and M2 as periplocin-related cardiac glycoside metabolites. The key ^1^H- and ^13^C-NMR data for M1 and M2 are summarized in Table 1. Full spectral data are provided in the Supporting Information (Figures S5–S11), and the chemical structures of the five analytes are shown in Figure 3.

Structural comparison of the five analytes revealed distinct modifications around the A/B-ring region of the cardenolide nucleus. Periplocin, periplocymarin, and periplogenin share a periplogenin-type aglycone bearing a C-5 hydroxy group, whereas the two urinary metabolites, gomphogenin (M1) and 17*α*-asclepioside (M2), lack this functionality. M1 was identified as a free aglycone with additional A-ring hydroxylation, while M2 retained C-3 glycosylation on a 5-dehydroxylated cardenolide scaffold. These structural differences provided a basis for comparing the known periplocin-related analytes with the two isolated urinary metabolites in the subsequent H/R-induced H9c2 cell injury model.

### 2.3. Cytotoxicity and Non-Cytotoxic Concentration Screening

**Figure 4:**
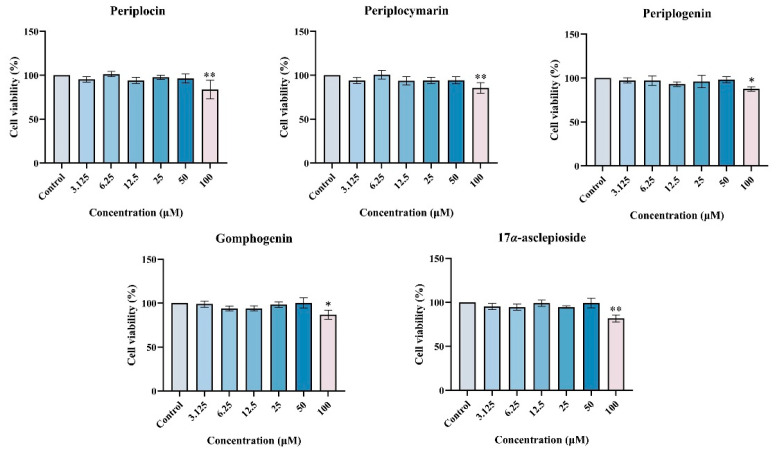
Effects of the metabolites on the viability of normal H9c2 cells (*n* = 3). Data are presented as the mean ± SD from three independent experiments. *: *p* < 0.05, **: *p* < 0.01 compared with the normal control group.

Before the H/R-induced injury assay, the cytotoxicity of the five periplocin-related analytes was evaluated in H9c2 cells. As shown in Figure 4, treatment with periplocin, periplocymarin, periplogenin, gomphogenin (M1), or 17*α*-asclepioside (M2) at 3.125–50 μM did not significantly reduce cell viability compared with the normal control group (*p* > 0.05). In contrast, exposure to 100 μM reduced cell viability to different extents (*p* < 0.05 or *p* < 0.01), indicating cytotoxicity at the highest tested concentration. Therefore, 25 and 50 μM were selected as the low and high non-cytotoxic concentrations for subsequent H/R-induced injury experiments.

### 2.4. Cytoprotective Effects of Periplocin-Related Analytes Against H/R-Induced Injury in H9c2 Cells

**Figure 5:**
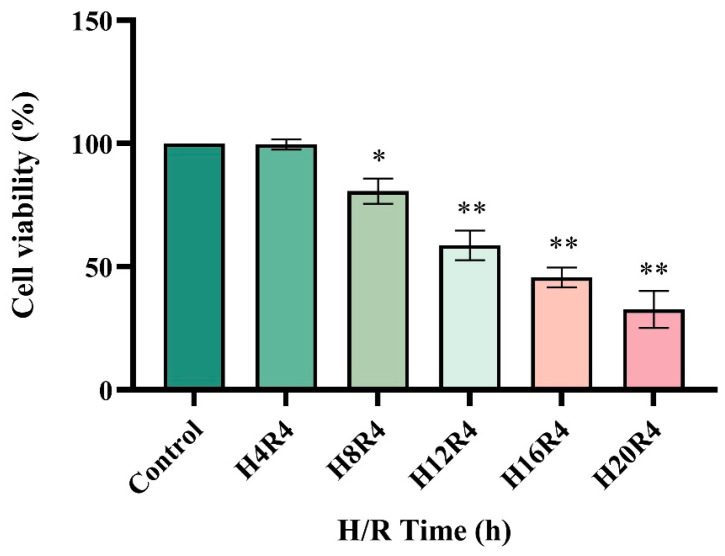
Effects of different H/R durations on the viability of H9c2 cells (*n* = 3). Data are presented as the mean ± SD from three independent experiments. Cells were subjected to hypoxia for 4, 8, 12, 16, or 20 h, followed by 4 h of reoxygenation (H4R4, H8R4, H12R4, H16R4, and H20R4, respectively). *: *p* < 0.05, **: *p* < 0.01 compared with the normal control group.

The H/R injury conditions were first optimized in H9c2 cells. As shown in Figure 5, H4R4 caused little change in cell viability compared with the normal control group, whereas cell viability progressively decreased as the hypoxia duration was extended. A significant reduction in viability was observed under H8R4, and viability decreased to approximately 60% of the control level under H12R4, while a sufficient dynamic range for detecting protective effects was retained. More severe injury was observed under H16R4 and H20R4 conditions. Therefore, H12R4 was selected as the working condition for the subsequent cytoprotective assay.

**Figure 6:**
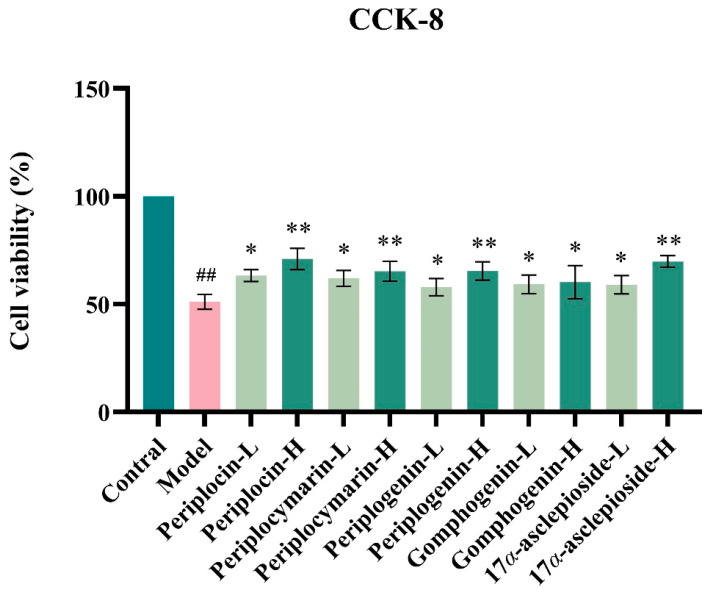
Effects of periplocin-related analytes on the viability of H/R-injured H9c2 cells (*n* = 3). Data are presented as the mean ± SD from three independent experiments. L and H indicate low and high non-cytotoxic concentrations, corresponding to 25 and 50 μM, respectively. ^##^: *p* < 0.01 compared with the normal control group. *: *p* < 0.05, **: *p* < 0.01 compared with the H/R model group.

Under the optimized H/R condition, cell viability was significantly decreased in the model group compared with the normal control group (*p* < 0.01), confirming successful establishment of the H/R-induced injury model (Figure 6). Compared with the H/R model group, pretreatment with periplocin, periplocymarin, periplogenin, gomphogenin (M1), or 17*α*-asclepioside (M2) significantly increased cell viability at both 25 and 50 μM (*p* < 0.05 or *p* < 0.01). For each analyte, the increase in cell viability was generally greater at 50 μM than at 25 μM. Notably, the two urinary metabolites isolated under diagnostic ion guidance, M1 and M2, also improved cell viability under the same experimental conditions. These results indicate that periplocin-related analytes, including the isolated urinary metabolites, showed preliminary viability-preserving effects in the H/R-injured H9c2 cell model.

Previous studies have implicated the steroid nucleus, C-17 butenolide ring, and C-3 substituent in cardenolide activity [31,32,33,34]. Under the present assay conditions, all five analytes increased the viability of H/R-injured H9c2 cells. This response was observed for the three C-5-hydroxylated analytes and for M1 and M2, which lack this group. Thus, within this limited compound set, the absence of C-5 hydroxylation did not eliminate the viability response. However, concurrent differences in C-3 glycosylation and A-ring oxygenation prevent attribution of the observed effects to a single structural feature. Moreover, the biological evaluation was based on CCK-8 cell-viability measurement in H9c2 cells, and 25 and 50 μM exceed typical in vivo exposure levels of cardiac glycosides. A positive cardioprotective control was also not included in the H/R assay. Therefore, the observed increases in cell viability should be regarded as preliminary in vitro evidence of biological activity, and further studies using positive reference compounds, complementary endpoints, physiologically relevant exposure levels, and more representative models are needed to clarify the biological relevance of these metabolites.

Compared with previous studies that mainly focused on LC-MS/MS detection or pharmacokinetic analysis of periplocin and its known metabolites, such as periplocymarin and periplogenin, the present study extends the workflow from metabolite screening to targeted isolation and structural confirmation. Recent studies have shown that diagnostic fragment ions, mass defect filtering, and LC-HRMS/MS-based data mining can improve the prioritization and annotation of structurally related metabolites in complex biological samples [35,36,37]. In addition, LC-MS- or LC-HRMS/MS-guided strategies have been increasingly used to support the targeted isolation of trace natural products from complex matrices. In the present study, the diagnostic ion at *m*/*z* 355.2270 was used as a class-informative marker for guiding candidate selection and purification, while final structural confirmation relied on HR-MS and NMR evidence. This diagnostic ion-guided strategy enabled the enrichment of trace periplocin-related metabolites from a complex urinary matrix and yielded isolated reference compounds that may support future pharmacokinetic, pharmacodynamic, and toxicological studies of periplocin-related cardenolides.

The retained activity of M1 and M2 is noteworthy from a structure-activity perspective. Previous studies have shown that the biological activity of cardiac glycosides is governed by coordinated contributions from the steroid nucleus, the C-17 butenolide ring, the C-14*β* hydroxy group, and the C-3 substituent or sugar moiety, rather than by a single structural feature [29,30]. At the receptor level, the steroid nucleus has been proposed to provide the core recognition framework for Na^+^/K^+^-ATPase inhibition, whereas the C-17 butenolide ring and C-3 sugar substituent contribute substantially to the overall interaction energy [29,30]. Three-dimensional SAR analysis further indicated that favorable interactions are mainly associated with the sugar and lactone regions, while additional polar substitution on the steroid core may have context-dependent or even unfavorable effects [31]. Consistently, cardenolide SAR analysis in cell-based models has suggested that C-3 substitution and overall lipophilicity/polarity are important determinants of activity, whereas C-5 hydroxylation alone is not necessarily decisive [32]. In the present study, the C-5-hydroxylated analytes periplocin, periplocymarin, and periplogenin increased the viability of H/R-injured H9c2 cells. Importantly, M1 and M2, which lack the C-5 hydroxy group, also retained cytoprotective activity under the same conditions. This pattern suggests that C-5 hydroxylation may modulate, but is not an absolute structural requirement for, the cytoprotective response observed in this H/R model. The retained activity of M1 and M2 may be related to the preservation of the cardenolide pharmacophore, particularly the C-14*β* hydroxy group and C-17 butenolide ring, together with differences in C-3 glycosylation and A-ring oxygenation. Because Na^+^/K^+^-ATPase binding, calcium handling, and downstream survival pathways were not directly measured, this interpretation should be regarded as a structure-based hypothesis rather than a confirmed mechanism.

## 3. Materials and Methods

### 3.1. Reagents, Chemicals, and Materials

Methanol and acetonitrile of chromatographic grade were purchased from Fisher Chemical (Fairlawn, OSU, USA). Formic acid of chromatographic grade was purchased from Anaqua Chemicals Supply (Wilmington, DE, USA). Methanol of analytical grade was purchased from Concord Technology Co., Ltd. (Tianjin, China). Ultrapure water was produced by a Milli-Q water purification system (Millipore, Milford, MA, USA). Periplocymarin, periplocin, and periplogenin were purchased from Chengdu Desite Bio-Technology Co., Ltd. (Chengdu, China). Periplocin was of analytical grade with a purity of ≥98%, and the same batch was used for oral administration in rats and as the analytical reference standard.

For cell experiments, Dulbecco’s Modified Eagle Medium (DMEM) with high glucose, penicillin–streptomycin solution (10,000 U/mL), and 0.25% trypsin-EDTA were purchased from Gibco Life Sciences (Grand Island, NY, USA). Fetal bovine serum (FBS) was obtained from BioInd Biological Industries (Kibbutz Beit HaEmek, Israel). Phosphate-buffered saline (PBS) was purchased from Solarbio (Beijing, China). The Cell Counting Kit-8 (CCK-8) was provided by MedChemExpress (Monmouth Junction, NJ, USA).

### 3.2. Characteristic Fragment Ion-Guided Screening of Periplocin Metabolites

#### 3.2.1. Urine and Plasma Collection

Male Sprague-Dawley rats (220 ± 10 g) were purchased from Beijing Huafukang Bio-Technology Co., Ltd. (Beijing, China). Before dosing, the rats were fasted for 12 h with free access to water. Periplocin was orally administered to 60 rats at 50 mg/kg as a suspension in sodium carboxymethylcellulose. The oral dose was selected based on our previous pharmacokinetic study of periplocin and its metabolites [26]. Treatment continued for one month in repeated six-day cycles comprising three consecutive dosing days followed by three drug-free days. This intermittent schedule was designed to increase the cumulative urinary recovery of low-abundance metabolites for preparative isolation without continuous drug exposure. All 60 rats were used for cumulative urine collection to obtain sufficient material for preparative isolation and structural characterization of trace metabolites. Urine samples were collected daily from all treated rats, stored at −80 °C, and subsequently combined into a single 13 L pool for preparative processing. This pooled urine sample served as the preparative source material. Among the 60 treated rats, six rats were randomly selected for serial plasma sampling after the first administration to confirm the presence of periplocin-related analytes in systemic circulation. Blood samples were collected from the orbital venous plexus via the medial canthus at 0.08, 0.25, 1, 2, 3, 4, 5, 6, 7, 8, 9, 10, 12, and 24 h after administration and transferred into heparinized tubes. Plasma was separated by centrifugation at 7000 rpm for 10 min and stored at −80 °C until analysis. Thus, plasma was used for initial metabolite screening, whereas urine was selected as the preparative matrix because its large cumulative volume provided sufficient material for enrichment, purification, and structural characterization. All animal procedures were approved by the Laboratory Animal Ethics Committee of Tianjin University of Traditional Chinese Medicine (approval no. TCM-LAEC2024032c1356) and performed in accordance with the committee’s guidelines.

The urine sample (1 mL) was deproteinized with 3 mL of acetonitrile. After vortex-mixing for 1 min and centrifugation at 14,000 rpm for 10 min, the supernatant was evaporated under a nitrogen stream until dry. The residue was redissolved in 200 μL of 50% methanol, vortex-mixed for 3 min, and then centrifuged for 10 min at 14,000 rpm. A 5 μL aliquot was injected into the HPLC-QQQ-MS/MS and UPLC-Q-TOF-MS/MS. The 100 μL plasma samples were extracted with 2 mL ethyl acetate, followed by centrifugation at 14,000 rpm for 10 min. The supernatant was dried under a flow of nitrogen gas. The residue was dissolved with 100 μL of 50% methanol, vortexed for 5 min, and centrifuged for 10 min at 14,000 rpm. Finally, 5 μL of the supernatant was injected into the HPLC-QQQ-MS/MS and UPLC-Q-TOF-MS/MS for analysis.

#### 3.2.2. HPLC-QQQ-MS/MS Condition

The Agilent 1200 HPLC system coupled to an Agilent 6430 triple-quadrupole mass spectrometer (Agilent Technologies, Santa Clara, CA, USA) were used for this work. A Waters XBridge^™^ BEH C18 column (4.6 mm × 50 mm, 2.5 μm. Milford, MA, USA) was used for chromatographic separation at a 30 °C thermostated column compartment. In the mobile phase, 0.1% formic acid aqueous solution and acetonitrile were used as solvents A and B, respectively. Gradient elution was performed as follows: 0–5 min, 5–26% B; 5–23 min, 26–65% B; 23–30 min, 65–95% B. The flow rate was set at 0.3 mL/min, and the injection volume was set at 5 μL.

Multiple reaction monitoring (MRM) was used for analysis in positive ESI mode. The MS parameters were designed as follows: drying gas was 300 °C; drying gas flow rate was 11 L/min; nebulizer was 15 psi; capillary voltage was 4 kV. In the HPLC-QQQ-MS/MS method, periplocin was monitored using a pseudo-MRM channel of *m*/*z* 719.4. Under positive-ion conditions, *m*/*z* 719.4 corresponded to the intact sodium adduct [M + Na]^+^. Both Q1 and Q3 were therefore set to *m*/*z* 719.4, and a low collision energy of 3 eV was applied to minimize collision-induced dissociation and permit transmission of the intact ion through the collision cell. Thus, this channel was used for intact-ion monitoring rather than as a conventional precursor-to-product MRM transition. The compound parameters are listed in Table 2.

The HPLC-QQQ-MS/MS procedure was used for the initial qualitative screening of blank and post-dose biological samples. The MRM settings for periplocin, periplocymarin, and periplogenin were adapted from a previously validated LC-MS/MS assay [15]. The intra-day precision, inter-day precision, and repeatability of these three analytes were evaluated using six replicate standard solutions and expressed as the relative standard deviations of the peak responses. At the screening stage, M1 and M2 were unassigned signals, and authentic standards were unavailable. They were therefore excluded from the calibration-based validation, and the QQQ data were not used for their quantitative determination.

#### 3.2.3. UPLC-Q-TOF-MS/MS and NMR Conditions

The UPLC-Q-TOF-MS/MS system comprises an Agilent 1290 Infinity UPLC (Agilent Technologies, Santa Clara, CA, USA) and an Agilent 6520 QTOF. Chromatographic separation was achieved on a Waters XBridge^™^ BEH C18 column (4.6 mm × 50 mm, 2.5 μm). The mobile phase containing 0.1% formic acid (A) and acetonitrile (B) was used to achieve the desired separation at a flow rate of 0.3 mL/min. The gradient program was as follows: 0–5 min, 5–26% B; 5–23 min, 26–65% B; 23–30 min, 65–95% B. The separation temperature was set at 35 °C, and the sample injection volume was 5 μL for analysis.

The mass spectrometer was operated in the positive dual-ESI mode. The MS parameters were designed as follows: capillary voltage, 5 kV; nebulizer, 55 psi; cone voltage, 80 V; drying gas (N_2_) flow rate, 11 L/min; collision energy, 20 V; and fragmentor voltage at 170 V with the temperature at 300 °C. Mass spectra were recorded across the range of *m*/*z* 0–2000. UPLC-Q-TOF-MS/MS provided accurate precursor-mass and product-ion data for evaluating the diagnostic fragments and associated neutral-loss pathways. It was also used to track target-containing fractions throughout purification.

NMR spectra were recorded at 298 K on Bruker AVANCE NEO 500 and 600 MHz spectrometers (Bruker BioSpin GmbH, Rheinstetten, Germany). The purified metabolites were dissolved in pyridine-*d5*. Chemical shifts are reported in parts per million (ppm) and were referenced to the residual solvent signals of pyridine-*d5* at δ_H_ 7.22 for ^1^H-NMR and δ_C_ 123.87 for ^13^C-NMR. Coupling constants (J) are reported in hertz. ^1^H-NMR, ^13^C-NMR, and ^1^H-^1^H NOESY spectra were acquired for M1, while ^1^H-NMR, ^13^C-NMR, HSQC, and HMBC spectra were acquired for M2.

### 3.3. Diagnostic Ion-Guided Isolation and Purification of Urinary Metabolites

Frozen rat urine (13 L) was thawed, filtered, and subjected to AB-8 macroporous resin column chromatography. The column was eluted successively with H_2_O, 70% ethanol, and 95% ethanol (*v/v*), with a total elution volume of 75 L. The 70% ethanol fraction was concentrated and loaded onto a Sephadex LH-20 column, which was eluted with 50% methanol to afford two target-containing fractions. During purification, each fraction was monitored by UPLC-Q-TOF-MS/MS based on the diagnostic fragment ion at *m*/*z* 355.2270 and the precursor ions of M1 and M2.

The two target-containing fractions were individually separated by ODS column chromatography using an H_2_O–methanol gradient from 0% to 100% methanol. Under UPLC-Q-TOF-MS/MS guidance, M1 and M2 were mainly enriched in fractions eluted with 45% and 60% methanol, respectively. The enriched fractions were subsequently subjected to a second Sephadex LH-20 chromatographic step and purified by preparative HPLC using methanol–H_2_O (1:1, *v*/*v*), yielding M1 (4 mg, purity 90.23%) and M2 (8 mg, purity 94.40%). Relative to the initial 13 L of pooled urine, these amounts corresponded to volume-normalized isolated yields of 0.31 and 0.62 mg/L, respectively. Based on a nominal cumulative periplocin dose of approximately 9.90 g, the purity-corrected amounts corresponded to approximate molar recoveries of 0.065% for M1 and 0.102% for M2. These estimates reflect the overall recovery across metabolite formation, urinary collection, and multistep purification.

### 3.4. Cell Culture

The rat embryonic cardiomyoblast cell line H9c2 was cultured in high-glucose DMEM supplemented with 10% FBS and 1% penicillin–streptomycin solution. The cells were maintained in a humidified incubator at 37 °C with 5% CO_2_. Upon reaching approximately 80% confluence, the cells were passaged using 0.25% trypsin.

### 3.5. Cytotoxicity Assay and Concentration Screening

The cytotoxicity of the isolated metabolites was evaluated using the CCK-8 assay. To determine the optimal non-cytotoxic concentration, cells were treated with various concentrations (3.125, 6.25, 12.5, 25, 50, and 100 μM) of the test compounds under normoxic conditions. The final concentration of DMSO in the culture medium was strictly controlled below 0.1%. After incubation, CCK-8 solution was added to each well, and the absorbance was measured at 450 nm using a Spark multimode microplate reader (Tecan Group Ltd., Männedorf, Switzerland).

### 3.6. Hypoxia/Reoxygenation (H/R) Model and Cytoprotective Evaluation

An in vitro hypoxia/reoxygenation (H/R)-induced myocardial injury model was established in H9c2 cells using an anaerobic jar and an AnaeroPack system Mitsubishi Gas Chemical Co., Inc. (Tokyo, Japan). The oxygen concentration was reduced to 0.1% within 1 h, while 5% CO_2_ was maintained. To optimize the injury conditions, the normal culture medium was replaced with glucose-free and serum-free medium, and H9c2 cells were exposed to hypoxia for 4, 8, 12, 16, or 20 h, followed by 4 h of reoxygenation in normal complete medium. Control cells were maintained under normoxic conditions throughout the experiment. Based on the model-screening results, hypoxia for 12 h followed by reoxygenation for 4 h (H12R4) was selected for subsequent cytoprotective evaluation. H9c2 cells were pretreated with periplocin, periplocymarin, periplogenin, gomphogenin (M1), or 17*α*-asclepioside (M2) at low and high non-cytotoxic concentrations (25 and 50 μM, respectively) before H/R exposure. Cell viability was then determined using the CCK-8 assay. Each treatment condition included six technical replicate wells in each independent experiment, and the experiment was independently repeated three times.

### 3.7. Statistical Analysis

Statistical analyses were performed using SPSS 18.0 (SPSS Inc., Chicago, IL, USA). Cell-viability data are presented as the mean ± SD from three independent experiments. Each condition included six technical replicate wells per experiment, which were averaged to obtain one value for each independent experiment (*n* = 3). Data were normalized to the corresponding control within each experiment. Multiple-group comparisons were performed using one-way analysis of variance followed by Dunnett’s multiple-comparisons test. The normal control group was used as the reference for the cytotoxicity and H/R-condition experiments, whereas the H/R model group was used for the treatment experiment. A value of *p* < 0.05 was considered statistically significant.

## 4. Conclusions

In summary, this study developed a diagnostic ion-guided strategy for the targeted screening, enrichment, and purification of in vivo periplocin metabolites using the characteristic steroid-core fragment ion at *m*/*z* 355.2270. By coupling UPLC-Q-TOF-MS/MS analysis with macroporous resin enrichment, Sephadex LH-20 chromatography, ODS chromatography, and preparative HPLC, two trace metabolites were isolated from large-volume rat urine and identified as gomphogenin (M1) and 17α-asclepioside (M2) by HR-MS and 1D/2D-NMR spectroscopy. To our knowledge, this is the first targeted isolation and structural confirmation of these two periplocin-related metabolites directly from rat biological fluids. In an H/R-induced H9c2 cell injury model, M1 and M2 increased cell viability at 50 μM, supporting further investigation of their biological properties. Within the limited compound set examined, M1 and M2 increased cell viability despite lacking C-5 hydroxylation, providing an initial observation for future structure–activity studies. This work provides a practical workflow for obtaining trace cardenolide metabolites from complex biological matrices and supplies reference compounds for future pharmacokinetic, pharmacodynamic, and toxicological studies of periplocin and *Cortex Periplocae*.

## Figures and Tables

**Table 1:** ^1^H- and ^13^C-NMR data for metabolites M1 and M2 in pyridine*-d5*.

Position	M1		M2	
	*δ*_C_ ^a^	*δ*_H_ ^b^ (*J* in Hz)	*δ*_C_ ^a^	*δ*_H_ ^b^ (*J* in Hz)
1	44.1		26.3	1.91, 2.14 (m)
2	74.6		26.7	1.79, 1.97 (m)
3	68.0	4.74 (m)	75.9	4.45 (s)
4	37.9		35.5	1.81, 2.08 (m)
5	31.1		40.1	
6	26.2		35.6	1.45, 1.88 (m)
7	32.1		24.3	1.82, 2.17 (m)
8	41.8		41.3	1.85 (m)
9	39.7		41.2	1.65 (m)
10	40.9		41.4	
11	22.1		22.2	1.32, 1.55 (m)
12	40.5		38.9	1.40, 1.64 (m)
13	50.5		50.1	
14	85.1		85.2	
15	33.6		33.3	2.04, 1.84 (m)
16	27.7		27.5	1.57, 2.87 (m)
17	51.8	2.51 (t)	52.1	2.81 (m)
18	16.6	1.06 (s)	16.4	1.04 (s)
19	17.2	1.16 (s)	19.2	1.12 (s)
20	176.4		174.7	
21	74.2	5.36 (d,18.0), 5.07 (d,18.0)	76.0	5.34 (d,18.0), 5.06 (d,18.0)
22	118.1	6.17 (s)	117.9	6.16 (s)
23	174.9		176.1	
1′			97.7	5.36 (d,7.8)
2′			73.9	3.59 (dd, 7.8, 2.2)
3′			68.7	4.32 (s)
4′			74.1	3.51 (dd, 9.4, 2.2)
5′			70.8	4.19 (m)
6′			17.4	0.98 (s)

^a^ Recorded at 125 MHz; ^b^ recorded at 600 MHz; coupling constants (J) are given in Hz, and chemical shifts are given in ppm.

**Table 2:** Mass spectrometry parameters of periplocin, periplocymarin, and periplogenin.

Compound	Precursor Ion (*m*/*z*)	Product Ion (*m*/*z*)	Fragmentor Voltage (V)	Collision Energy (eV)	Ionization Mode
periplocin	719.4	719.4	135	3	Positive
periplocymarin	535.5	113.1	135	20	Positive
periplogenin	391.3	355.2	135	10	Positive

## Data Availability

The data presented in this study are available in the article and Supplementary Materials. Additional data are available from the corresponding author upon reasonable request.

## Supplementary Materials

The following supporting information can be downloaded at: https://www.mdpi.com/article/10.3390/molecules31142436/s1, Figure S1: The BPC and EIC chromatograms of the 70% ethanol fraction eluted from the AB-8 macroporous resin. (A) Base peak chromatogram (BPC) of the 70% ethanol fraction. (B) Extracted ion chromatogram (EIC) of target metabolite 1 (M1) in the 70% ethanol fraction. (C) EIC of target metabolite 2 (M2) in the 70% ethanol fraction. Peak 1: M1 (*m*/*z* 391.2452); Peak 2: M2 (*m*/*z* 521.3114); Figure S2: The BPC and EIC chromatograms of fractions enriched by the first Sephadex LH-20 column chromatography. (A) BPC of the M1-enriched fraction. (B) EIC of M1 in the M1-enriched fraction. (C) BPC of the M2-enriched fraction. (D) EIC of M2 in the M2-enriched fraction. Peak 1: M1 (*m*/*z* 391.2452); Peak 2: M2 (*m*/*z* 521.3114); Figure S3: The BPC and EIC chromatograms of fractions enriched by ODS open column chromatography. (A) BPC of the M1-enriched fraction. (B) EIC of M1 in the M1-enriched fraction. (C) BPC of the M2-enriched fraction. (D) EIC of M2 in the M2-enriched fraction. Peak 1: M1 (*m*/*z* 391.2452); Peak 2: M2 (*m*/*z* 521.3114); Figure S4: The BPC and EIC chromatograms of fractions purified by the second Sephadex LH-20 column chromatography. (A) BPC of the M1-purified fraction. (B) EIC of M1 in the M1-purified fraction. (C) BPC of the M2-purified fraction. (D) EIC of M2 in the M2-purified fraction. Peak 1: M1 (*m*/*z* 391.2452); Peak 2: M2 (*m*/*z* 521.3114); Figure S5: The ^1^H-NMR spectrum of M1; Figure S6: The ^13^C-NMR spectrum of M1; Figure S7: The ^1^H-^1^H NOESY spectrum of M1; Figure S8: The ^1^H-NMR spectrum of M2; Figure S9: The ^13^C-NMR spectrum of M2; Figure S10: The HSQC spectrum of M2; Figure S11: Key HMBC evidence for the structural assignment of M2. (A) HMBC spectrum of M2 recorded in pyridine-d5. (B) Selected HMBC correlations mapped onto the structure of M2; Table S1: Intra-day precision, inter-day precision, and repeatability of the HPLC-QQQ-MS/MS method for periplocin, periplocymarin, and periplogenin (*n* = 6).
