# Supplementary material for: Diagnostic Ion-Guided Isolation and Characterization of Trace Periplocin-Derived Cardenolide Metabolites from Rat Urine
Source: Molecules. 2026 Jul 11;31(14):2436. doi: 10.3390/molecules31142436 (PMC13415648; doi:10.3390/molecules31142436)
Supplement: Supplementary file 1 [file molecules-31-02436-s001.zip › molecules-4408488-supplementary.pdf]

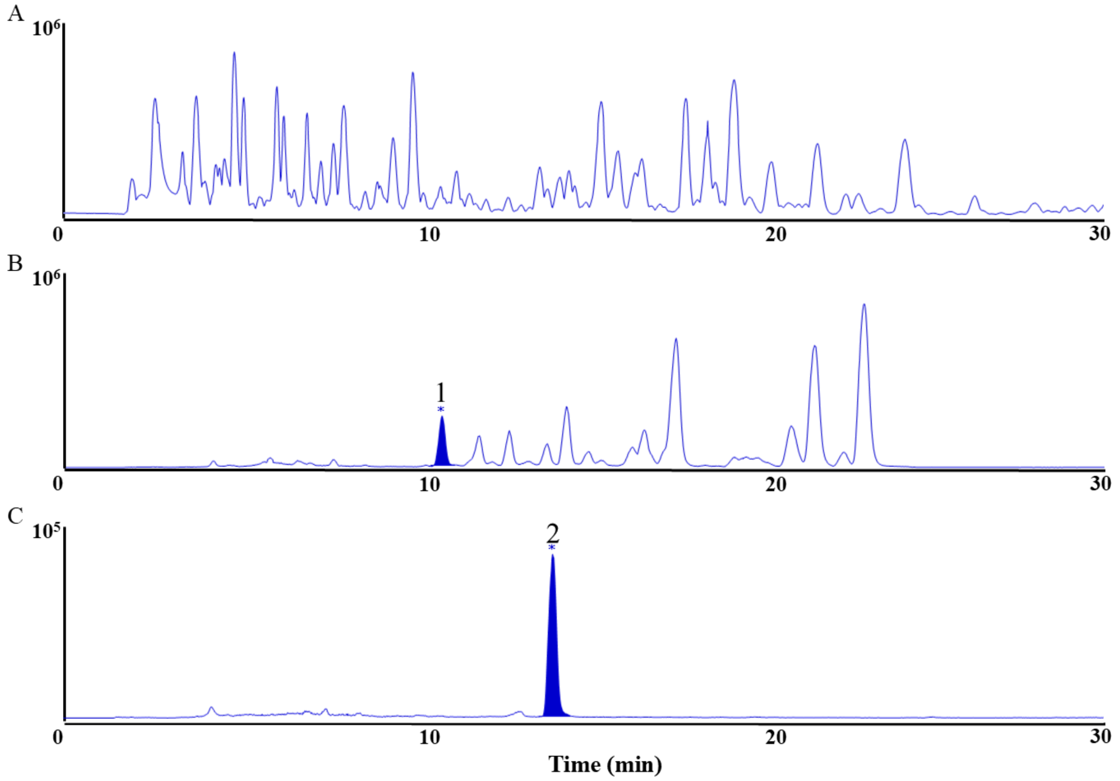

Figure S1 The BPC and EIC chromatograms of the 70% ethanol fraction eluted from the AB-8 macroporous resin. (A) Base peak chromatogram (BPC) of the 70% ethanol fraction. (B) Extracted ion chromatogram (EIC) of target metabolite 1 (M1) in the 70% ethanol fraction. (C) EIC of target metabolite 2 (M2) in the 70% ethanol fraction. Peak 1: M1 (*m/z* 391.2452); Peak 2: M2 (*m/z* 521.3114).

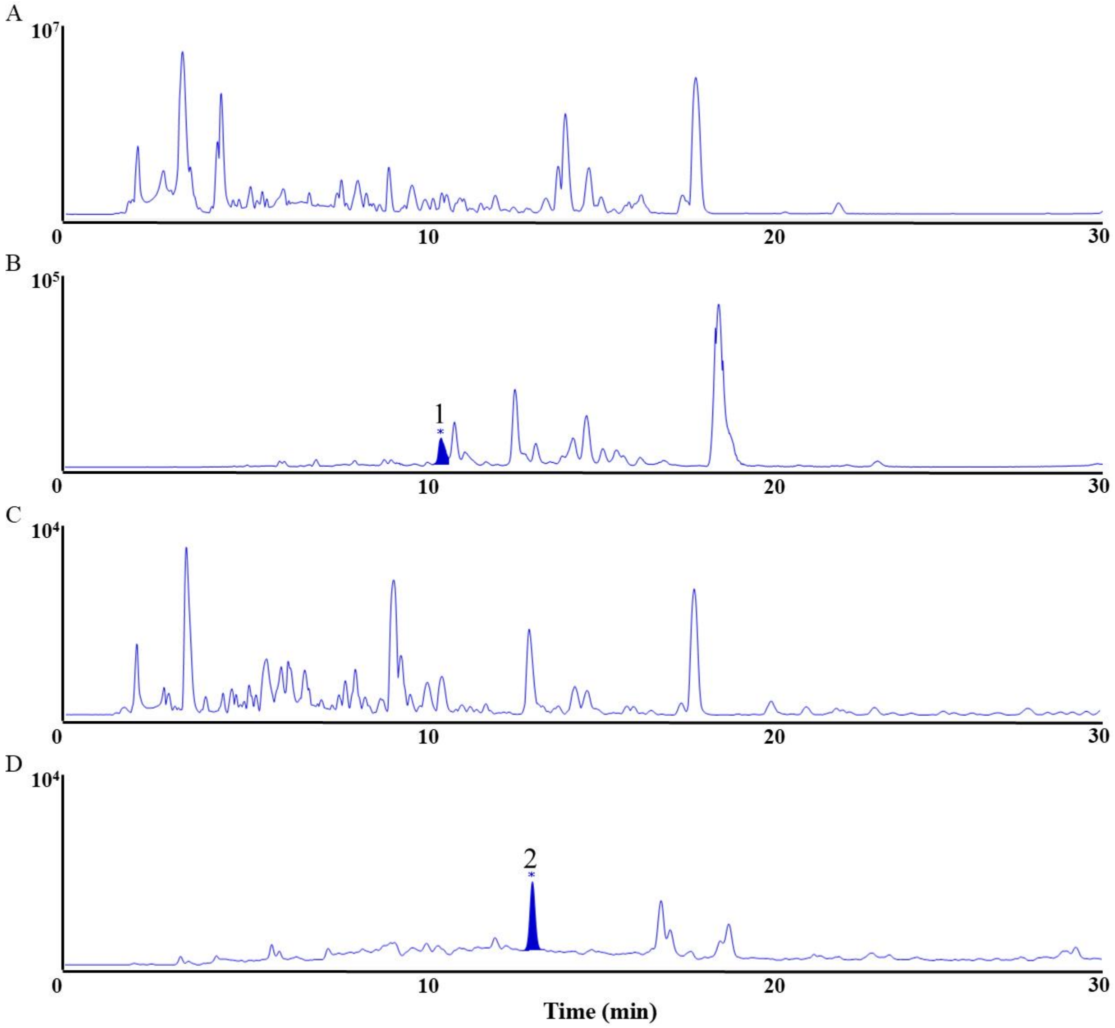

Figure S2 The BPC and EIC chromatograms of fractions enriched by the first Sephadex LH-20 column chromatography. (A) BPC of the M1-enriched fraction. (B) EIC of M1 in the M1-enriched fraction. (C) BPC of the M2-enriched fraction. (D) EIC of M2 in the M2-enriched fraction. Peak 1: M1 (*m/z* 391.2452); Peak 2: M2 (*m/z* 521.3114).

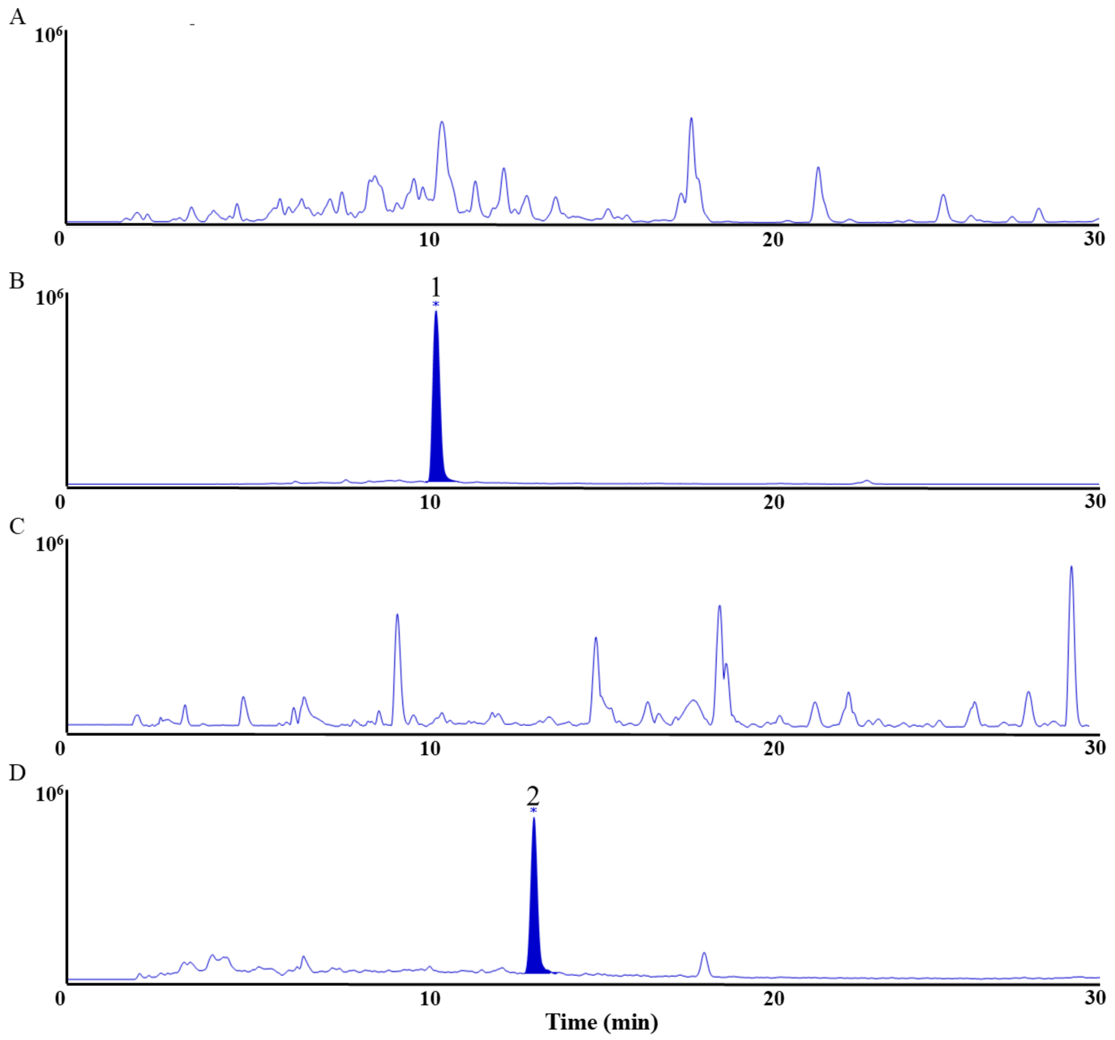

Figure S3 The BPC and EIC chromatograms of fractions enriched by ODS open column chromatography. (A) BPC of the M1-enriched fraction. (B) EIC of M1 in the M1-enriched fraction. (C) BPC of the M2-enriched fraction. (D) EIC of M2 in the M2-enriched fraction. Peak 1: M1 (*m/z* 391.2452); Peak 2: M2 (*m/z* 521.3114).

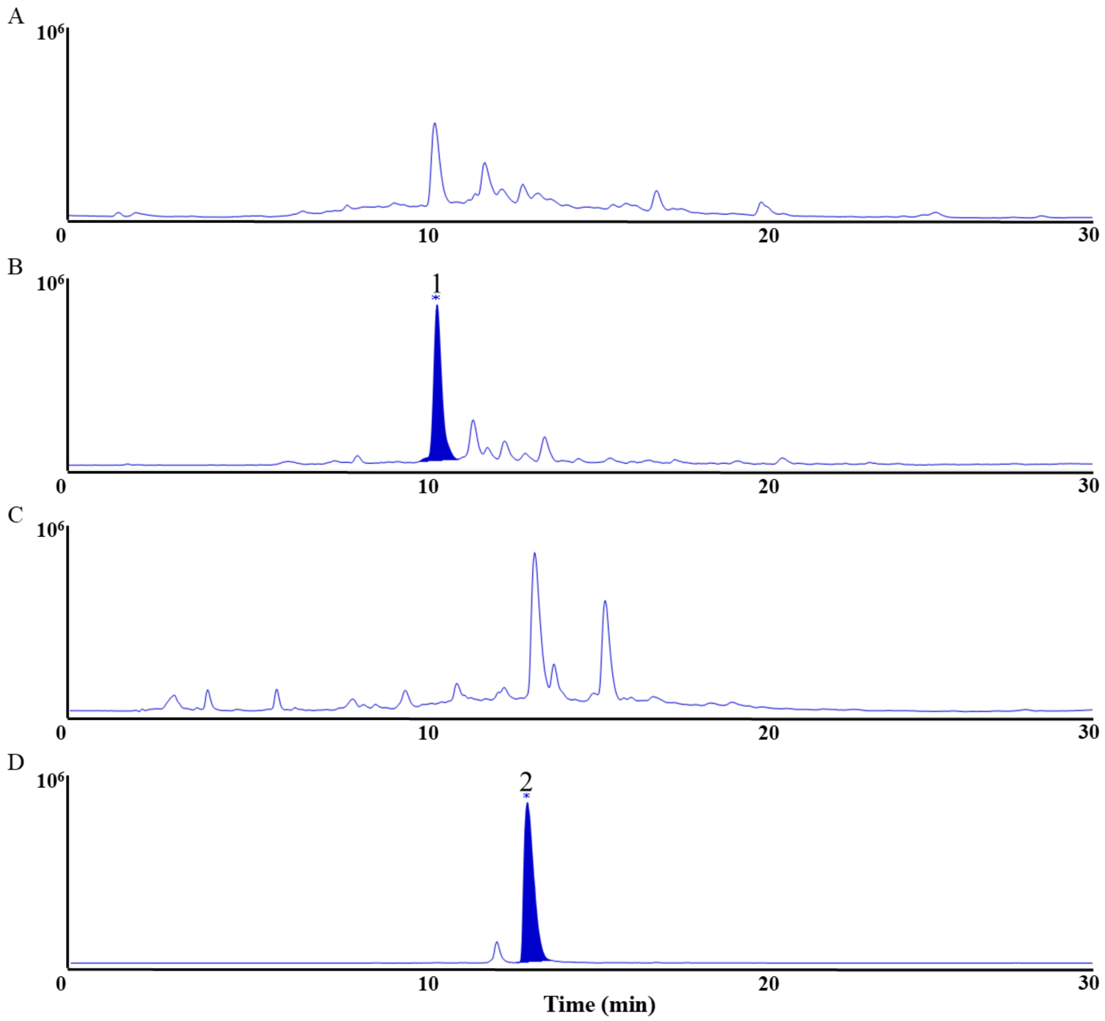

Figure S4 The BPC and EIC chromatograms of fractions purified by the second Sephadex LH-20 column chromatography. (A) BPC of the M1-purified fraction. (B) EIC of M1 in the M1-purified fraction. (C) BPC of the M2-purified fraction. (D) EIC of M2 in the M2-purified fraction. Peak 1: M1 (*m/z* 391.2452); Peak 2: M2 (*m/z* 521.3114).

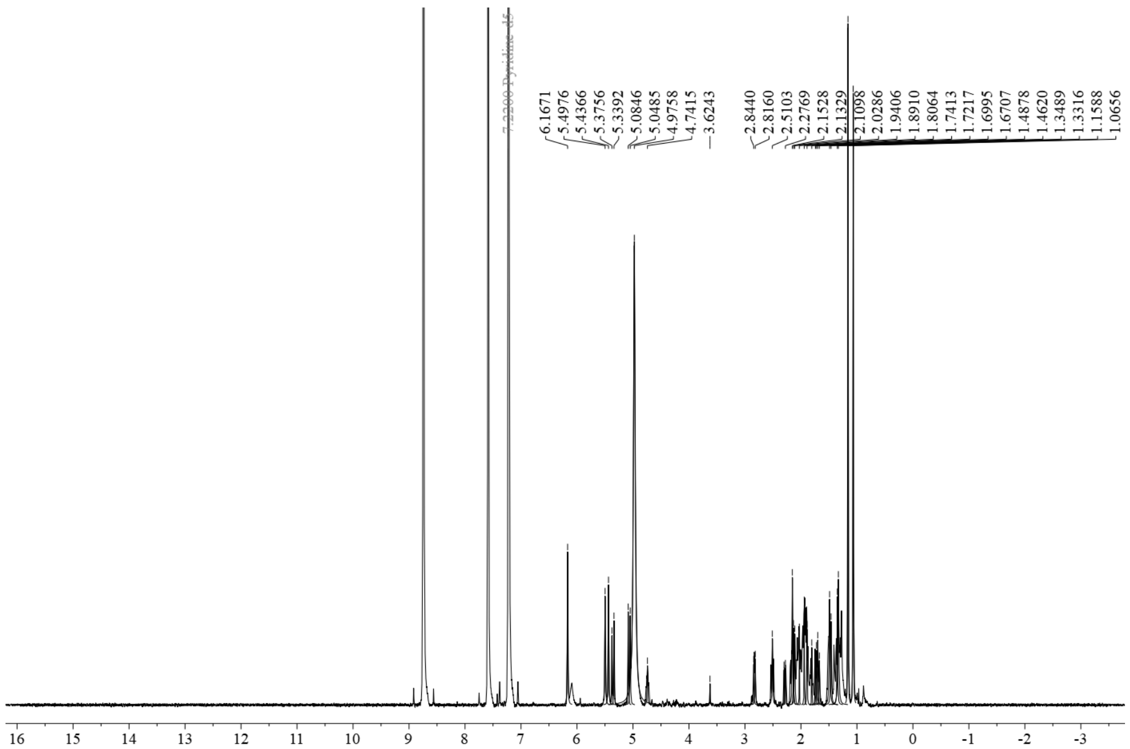

Figure S5 The ^{1}H-NMR spectrum of M1.

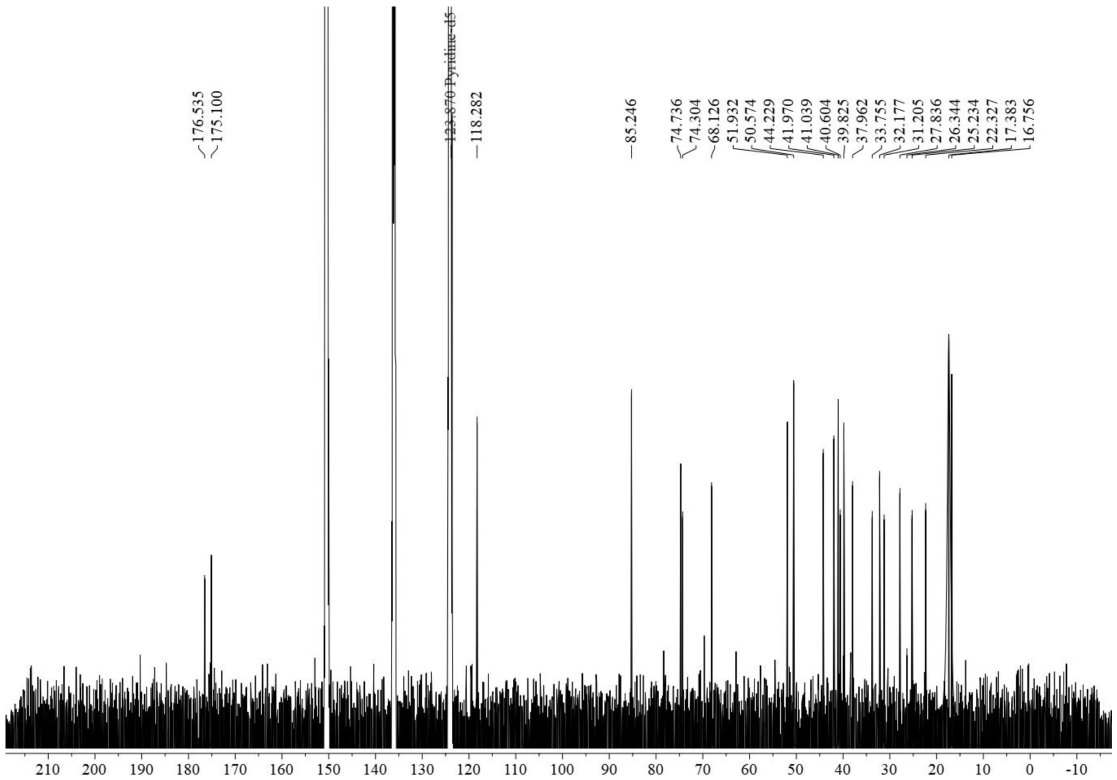

Figure S6 The ^{13}C-NMR spectrum of M1**.**

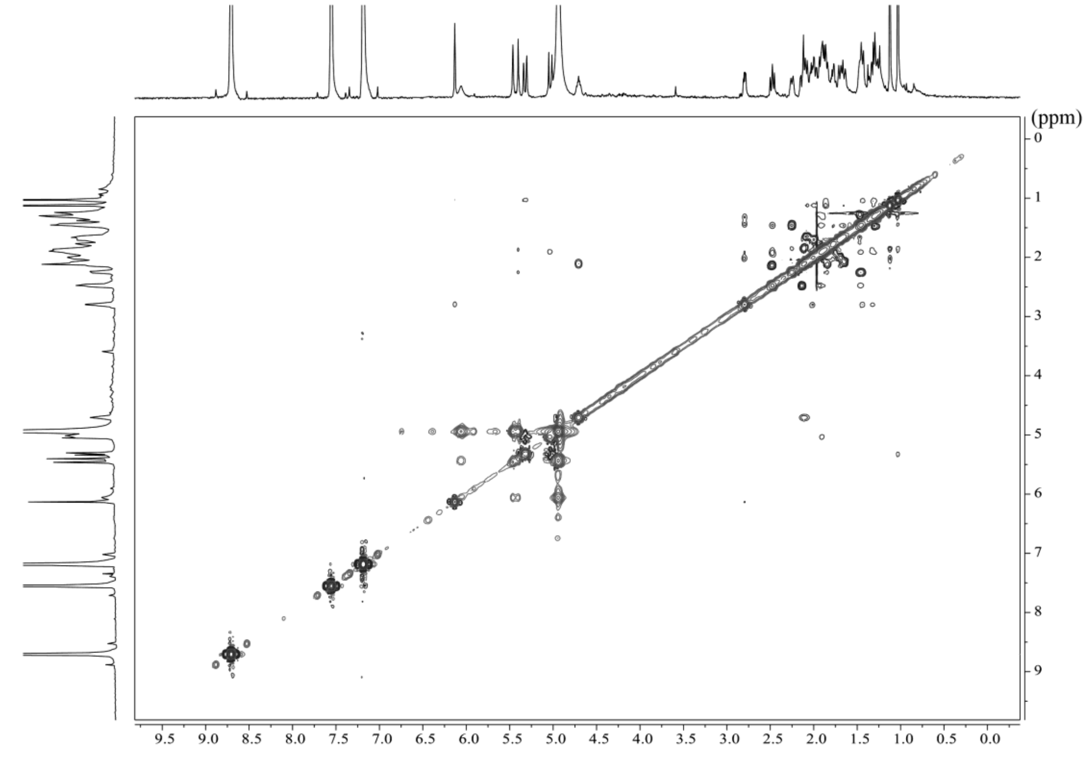

Figure S7 The ^{1}H-^{1}H NOESY spectrum of M1**.**

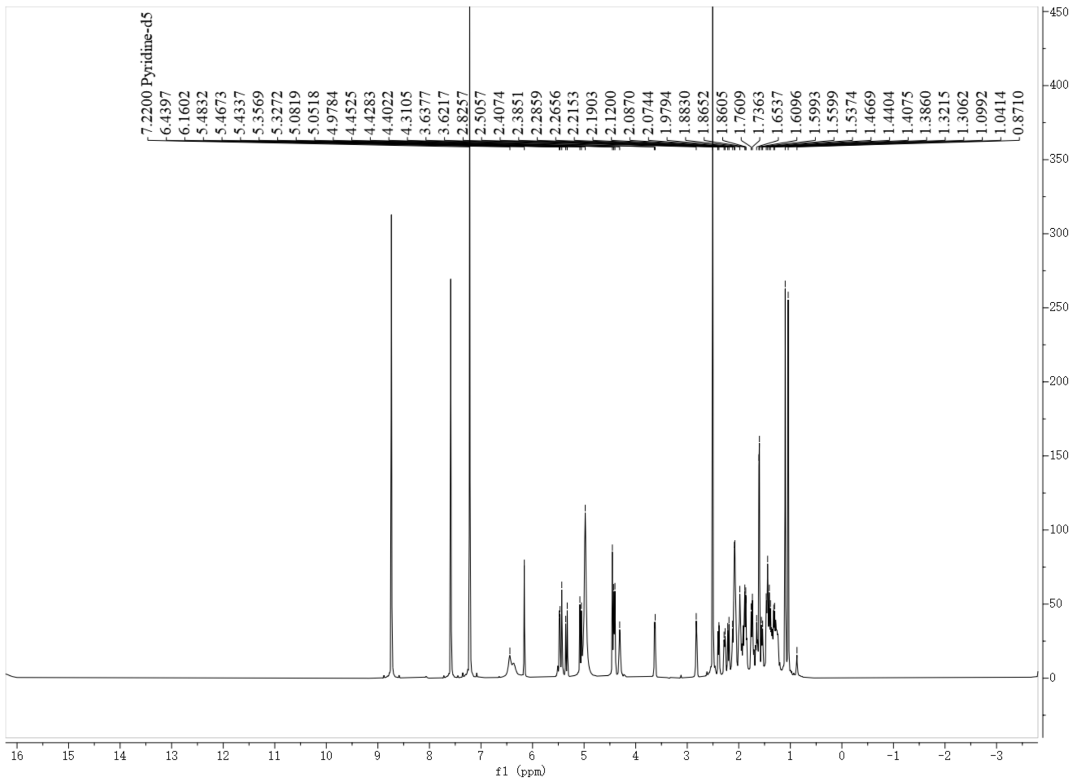

Figure S8 The ^{1}H-NMR spectrum of M2**.**

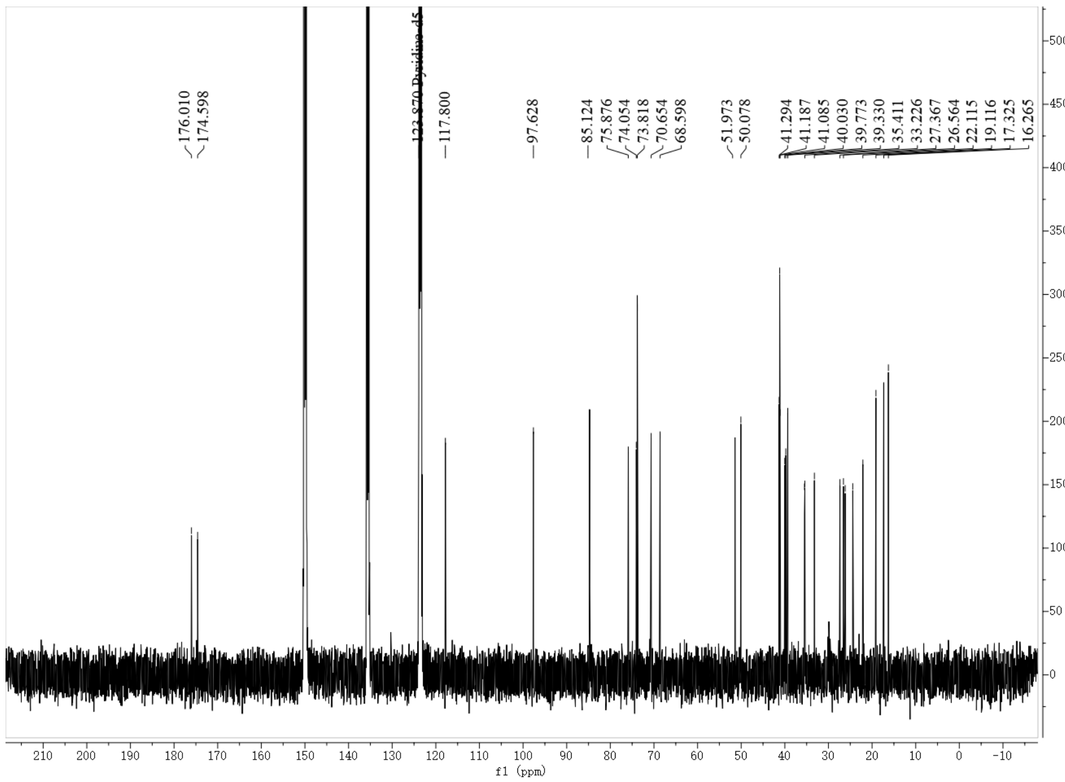

Figure S9 The ^{13}C-NMR spectrum of M2**.**

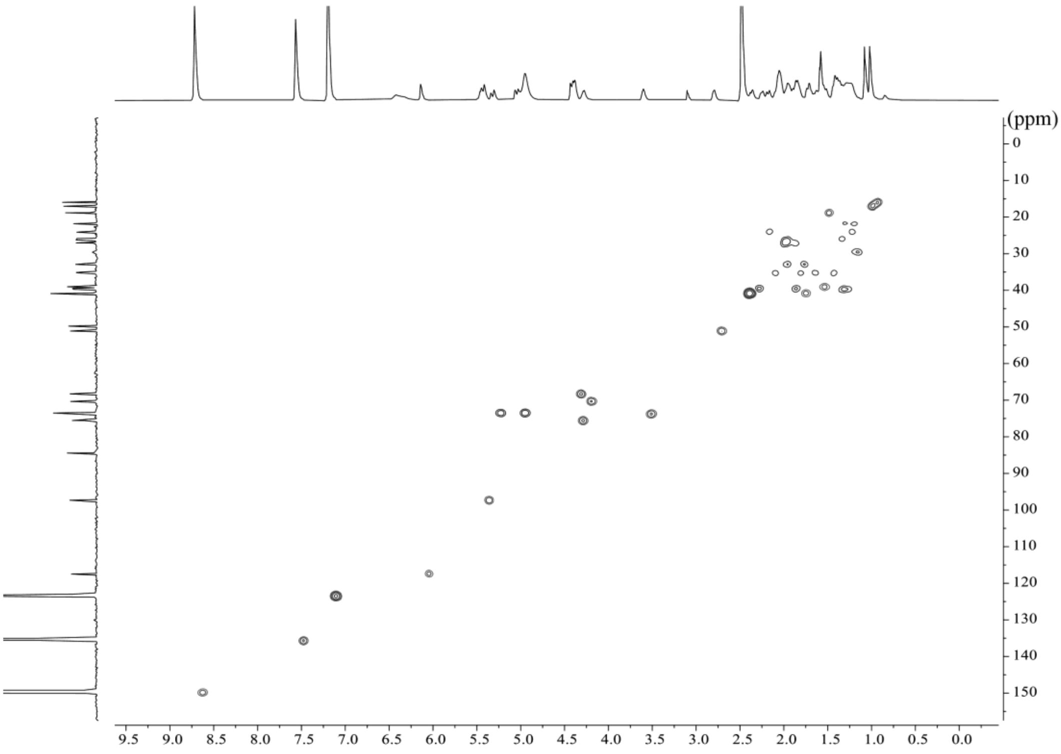

Figure S10 The HSQC spectrum of M2**.**

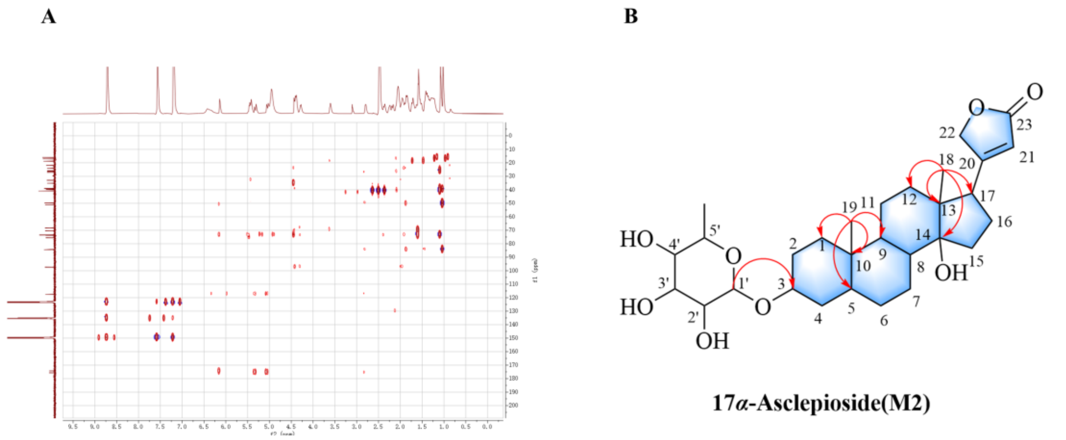

17α-Asclepioside(M2)

Figure S11 Key HMBC evidence for the structural assignment of M2. (A) HMBC spectrum of M2 recorded in pyridine-d5. (B) Selected HMBC correlations mapped onto the structure of M2.

Table S1 Intra-day precision, inter-day precision, and repeatability of the HPLC-QQQ-MS/MS method for periplocin, periplocymarin, and periplogenin (n = 6).

Compound	Precision RSD (%)		Repeatability
	Intra-Day	Inter-Day	RSD (%)
periplocin	5.23	4.81	2.33
periplocymarin	2.01	2.42	3.95
periplogenin	3.28	4.47	2.76
